# LncRNA-MIAT promotes thyroid cancer progression and function as ceRNA to target EZH2 by sponging miR-150-5p

**DOI:** 10.1038/s41419-021-04386-0

**Published:** 2021-11-22

**Authors:** Kai Guo, Kai Qian, Yuan Shi, Tuanqi Sun, Zhuoying Wang

**Affiliations:** 1 Department of Head and Neck Surgery, Shanghai Jiao Tong University School of Medicine Affiliated Renji Hospital, 200001 Shanghai, China; 2 Department of Head and Neck Surgery, Fudan University Shanghai Cancer Center, Shanghai, China; 3 Department of Oncology, Fudan University Shanghai Medical College, 200032 Shanghai, China

**Keywords:** Thyroid cancer, Tumour biomarkers

## Abstract

While long noncoding RNAs (lncRNAs) have been reported to play an important role in human cancer types, they remain poorly understood in papillary thyroid carcinoma (PTC). The aim of this study was to use genome-wide expression profiling to identify lncRNAs acting as competing endogenous RNAs (ceRNAs) in PTC. We constructed a ceRNA network based on our lncRNA microarray data and validated the correlation between myocardial infarction-associated transcript lncRNA (MIAT), miRNA-150-5p, and EZH2 in vitro and in vivo. We found 15 lncRNAs, 28 miRNAs, and hundreds of mRNAs involved in this ceRNA network. Splendid positive correlations were found between the MIAT and EZH2 expression in types of cancer in TCGA data. Besides, significant differences in MIAT/EZH2 expression were found among various clinicopathological features. Gain- and loss-of-function experiments revealed that MIAT inhibited cell proliferation and migration in vitro. Moreover, EZH2 was identified as a direct downstream target of miR-150-5p in PTC cells. Restoration of EZH2 expression partially abolished the biological effects of miR-150-5p. Furthermore, overexpression of MIAT was inversely correlated with miR-150-5p expression. Knockdown of MIAT produced significant behavioral alter maybe partly due to the function of the MIAT-150-5p-EZH2 network. Our findings suggest MIAT may inhibit EZH2 expression and promote PTC cell invasion via the miR-150/EZH2 pathway. Therefore, MIAT may serve as a valuable prognostic biomarker and therapeutic target for PTC.

## Introduction

Papillary thyroid carcinoma (PTC) is the most common endocrine malignancy and has a rapidly increasing global incidence rate [1]. Although most patients with PTC exhibit excellent survival, recurrence rates from 8 to 23% after the initial surgical treatment have been reported, which greatly impacts patient quality of life [2]. In recent years, studies have demonstrated that inappropriate gene expression because of genomic instability or epigenetic marker alterations plays a major role in the regulation of PTC [3–6]. Among other theories, the competing endogenous RNA (ceRNA) hypothesis has attracted more attention [7–9]. This hypothesis describes a complex post-transcriptional regulatory network mediated by microRNAs (miRNAs): by sharing one or more miRNA response elements (MREs), protein-coding, and long noncoding RNAs (lncRNAs) compete for binding to miRNAs and hence functionally liberate mRNA regulated by the miRNAs. However, to understand the function of ceRNA networks in the pathogenesis and pathological conditions of PTC, further research is required.

In this study, we hypothesized that there were still a significant number of previously unexplored genetic differences between PTC and normal thyroid tissues, especially with respect to lncRNA expression patterns. We analyzed paired PTC tumor and noncancerous tissue samples to profile the differentially expressed lncRNAs by lncRNA microarray analysis. We then collected miRNA and mRNA expression data from public databases to construct a lncRNA–microRNA–mRNA regulatory network. Critical lncRNAs were further identified by correlation with the clinical characteristics and were validated by experiments in vitro and in vivo.

## Materials and methods

### Patients, samples, and ethics statement

Sixty-four PTC tumor and paired adjacent noncancerous thyroid tissue specimens were obtained from the Department of Head and Neck Surgery, Shanghai Jiao Tong University School of Medicine Affiliated Renji Hospital. The sample size was selected according to the published literature and matched according to 1:1. Eligibility criteria included: (a) PTC proved by post-operative pathology report (FTC and MTC patients were not included); (b) initial treatment at our hospital with no previous locally curative surgery or radiation therapy of the head or neck. All tissue samples were snap-frozen in liquid nitrogen immediately after thyroidectomy and were transferred to the freezer at −80 °C before use. Four sample pairs were used for the microarray study. The other sixty sample pairs were used for qRT-PCR validation. All of the tissue specimens were obtained for this study with informed consent from the patients, and the use of the human specimens was approved by the Institutional Ethics Committee of Renji Hospital. All procedures performed in our study were consistent with the ethical standards of our institutional research committee.

### Expression microarray and bioinformatics analysis

The microarray (SBC Human lncRNA microarray v6.0, Shanghai Biotechnology Corporation, Shanghai, China) used in this study could detect 77,103 lncRNAs, including GENCODE v21/Ensembl (18,100), LNCipedia v3.1 (49,328), LncRNAdb (32), Noncoder v4 (10,079), and the University of California Santa Cruz genome browser (UCSC, 25,919). Preprocessing of the public microarray data was conducted using the R software environment and packages from the Bioconductor project. Raw data were collected from CEL files and preprocessed with the robust multichip average (RMA) algorithm for background correction, quantile normalization, and median polish summarization. After RMA preprocessing, a set of probe ID-centric gene expression values were available for downstream analysis.

### RNA extraction and quality assessment

Total RNA was extracted from frozen tissue samples and fresh cultured cells using RNAiso Plus (TaKaRa, Dalian, China) according to the manufacturer’s instructions. The quantity of RNA was determined using a NanoDrop 2000 spectrophotometer (Thermo Scientific, Wilmington, DE, USA). The ratio of the absorbances at 260 and 280 nm (2.0 ≥ A260/A280 ≥ 1.8) was used to assess RNA purity.

### RNA reverse transcription and qRT-PCR

High-quality total RNA was then reverse-transcribed using Prime Script RT Master Mix (TaKaRa, Dalian, China) following the manufacturer’s instructions. qRT-PCR was performed using SYBR Premix Ex Taq II (TaKaRa, Dalian, China) on a Light Cycler 480 system (Roche) following the manufacturer’s instructions. The amplification conditions were 95 °C, for 5 s and 60 °C for 45 s, with a total of 40 cycles. Glyceraldehyde 3-phosphate dehydrogenase (GAPDH) was used as an internal control. The primers were synthesized by TaKaRa Bio (Dalian, China).

### Construction of the ceRNA network

Construction of the ceRNA network included three steps: (i) lncRNAs that were up- or downregulated with a fold change of 5.0 and a *P* value less than 0.01 were collected; (ii) lncRNA–miRNA interactions were predicted by miRcode (http://www.mircode.org/); and (iii) mRNAs that were targeted by miRNAs with experimental support were identified using TarBase (http://www.microrna.gr/tarbase). The lncRNA–miRNA–mRNA regulatory network was constructed with the above interactions. The network was visualized using Cytoscape software (http://cytoscape.github.io/).

### Cell culture and cell transfection

In total, three cell types were used in this study: NTHY, TPC-1, and K1 cells which were purchased from the ATCC cell bank. NTHY and TPC-1 cells were grown in RPMI 1640 medium supplemented with 10% fetal bovine serum (FBS). K1 cells were cultured in DMEM containing 10% FBS. The cells were maintained at 37 °C in a humidified atmosphere containing 5% CO_2_.

For transfection, cells were seeded in 6-well plates at a concentration of 2 × 10^5^ cells/well. When the cells reached 40–60% confluence, they were transfected with corresponding plasmids. MIAT interfering plasmid pLVX-MIAT-short hairpin RNA (MIAT-shRNA) was aimed to downregulate MIAT expression. A scrambled sequence was inserted into pLVX-shRNA1 plasmid, which was used as the control. MIAT overexpression plasmid pcDNA3.1-MIAT was used to upregulate MIAT expression. miR-150-5p mimic, miR-150-5p antagomir (anti-miR-150-5p), and their controls were designed and synthesized by BioSune Biotechnology Co., Ltd. (Shanghai, China). Both PTC cell lines were transfected with the above vectors using Lipofectamine 2000 reagent (Invitrogen, Karlsruhe, Germany) following the manufacturer’s instructions.

### Western blot

Cells were lysed in radioimmunoprecipitation assay buffer (Sigma) supplemented with a protease inhibitor cocktail (Roche). Equal amounts of protein extract were loaded onto gels for 10% sodium dodecyl sulfate-polyacrylamide gel electrophoresis. Then, the proteins were transferred to a polyvinylidene fluoride membrane (Millipore, Billerica, MA, USA) and incubated in skimmed milk for 1 h at room temperature. The membrane was incubated with indicated primary antibodies at 4 °C overnight. The membrane was incubated with goat anti-rabbit IgG (Abcam) and then visualized using an ECL Western Blot Kit (CWBIO). Protein levels were quantified using Image J software (NIH, Bethesda, MD, USA). After the background was subtracted, the signal of each target band was normalized to that of the β-actin band. The antibody information is shown in Supplementary Table 1.

### Cell proliferation assay, flow cytometry analysis of cell cycle and apoptosis, and migration assay

The cell proliferation was investigated by Cell Counting Kit 8 (CCK-8, Dojindo, Japan) assay according to the manufacturer’s protocol. Approximately 2000 cells/well were seeded into 96-well plates, cultured for 12 h, and then transfected with MIAT-shRNA, pcDNA3.1-MIAT or the negative control for 24 h. For the cell cycle analysis, 1 × 10^6^ cells were seeded into six-well plates. After 24 h of transfection, the cells were collected and fixed in chilled 70% ethanol at −20 °C for 12 h or overnight, followed by washing with phosphate-buffered saline. The fixed cells were stained with 50 mg/ml propidium iodide (PI) at room temperature for 20 min. For the apoptosis assay, the cells were stained with annexin V-FITC/PI (MultiScience Biotech, Hangzhou, China). The flow cytometry analysis was performed using a FACSCalibur system (BD Biosciences, USA). The results were analyzed using MODFIT (version 4.1) and FlowJo software (version 7.6). The results of each assay are representative of three independent experiments performed in triplicate. Cell migration ability was measured using a transwell chamber (8-μm pore size, Corning Costar, USA). For transwell assay, 10 × 5 cells suspended in serum-free DMEM medium were seeded evenly into the upper chamber and the lower chamber contained medium supplemented with 20% FBS. After 30 h incubation, the filters were fixed in methanol and stained with 0.5% crystal violet. The upper faces were gently abraded, and the lower faces were imaged and counted under the microscope.

### Luciferase reporter assay

MIAT cDNA was amplified from human PTC tissues and inserted into pGL3 plasmid. Also, the mutant miR-150-5p binding sequence was introduced to construct the MIAT mutant plasmid. TPC-1 cells were seeded into 48-well plates, followed by incubation for 24 h. Then, cells were transfected with MIAT-wt or MIAT-mut plasmid in combination with miR-150-5p mimics, control mimics, and pGL3-EZH2 3’-UTR wild type or mutant using Lipofectamine 3000. After transfection for 48 h. The relative luciferase activities were measured using the Dual-Luciferase Reporter Assay System (Promega, Madison, WI, USA) and normalized to Renilla activity.

### Animal treatment

BALB/c mice (female, 4–6 weeks of age, 18–20 g; Shanghai SLAC Laboratory Animal Co., Ltd.) were housed under pathogen-free conditions and given food and acidified water ad libitum. Ten mice were randomized in a blinded fashion into two groups (5 mice/group) and assigned to different treatments. The sample size was determined based on statistical power and ethical guidelines. Criteria for animal inclusion and exclusion were based on an independent assessment according to AAA-LAC guidelines. The mice of each group were injected subcutaneously with 2.0 × 10^7^ TPC-1 and TPC-1-MIAT-shRNA cells in 100 μL PBS. All animal experiments were performed according to the Guidelines for the Care and Use of Laboratory Animals and were approved by the Institutional Animal Care and Use Committee of Shanghai Jiaotong University.

### Statistical analysis

All the experimental data were analyzed using Stata (version 12.0; Stata Corp, College Station, TX). Continuous variables are described using the mean ± SD, and the differences were calculated using an unpaired Student *t* test. Categorical variables are described using the frequency and percent and were compared using the chi-squared test or Fisher exact test. The linear correlation between the expression levels of TCGA genes involved in this study was drawn by using the *scatter* and *lfit* commands and calculated by *reg* command in Stata. Survival probabilities were calculated using the Kaplan–Meier method. Differences in survival rates were tested with the log-rank test. All the reported *P* values are two-sided, and *P* < 0.05 was considered significant.

## Results

### Differentially expressed lncRNAs in PTC tumor tissue samples

**Fig. 1:**
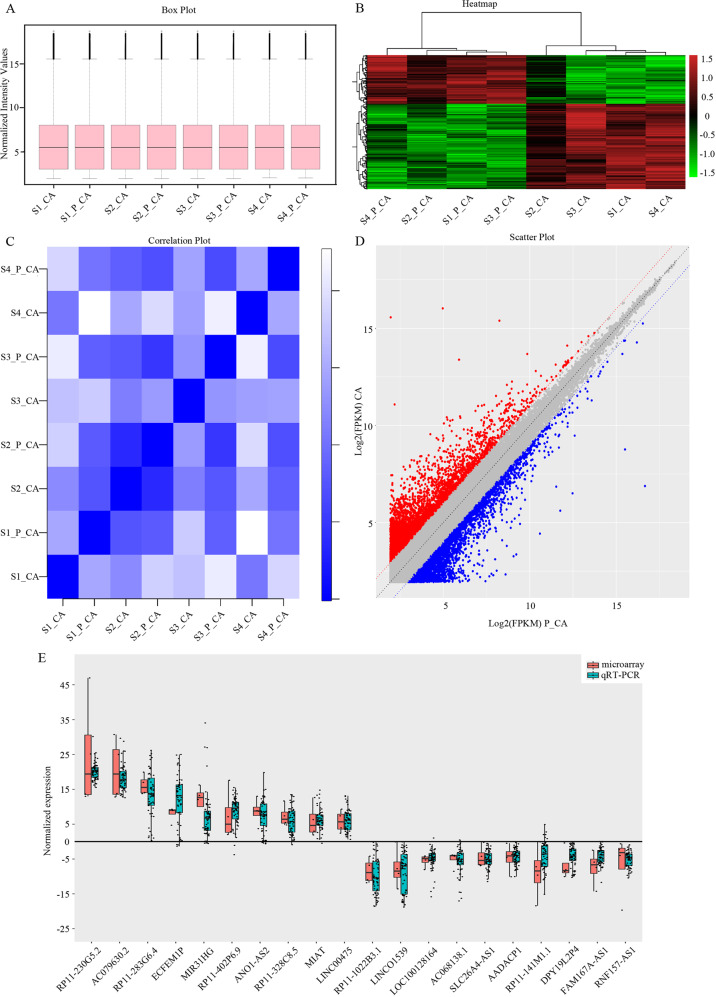
Quality control and gene scattering of lncRNA microarray. **A** Each box represents a sample plotted by the sample name on the horizontal axis and log2 (value of the probe signal) on the vertical axis. The upper and lower sides of the rectangular box correspond to the first and third quartiles (Q1 and Q3), while the line in the rectangular box represents the median. The upper line indicates Q1 + 1.5IQR, and the lower line indicates Q3-1.5IQR, where IQR represents the interquartile range for the four-point range, IQR = Q3-Q1. **B** Heatmap demonstrating the expression of lncRNAs in the different samples. Red indicates a higher relative expression, while green indicates a lower relative expression. **C** Correlation analysis between normal thyroid and PTC tissues. Cross-correlation coefficient figure mapping according to the correlation coefficient between different samples; the deeper the color, the stronger the correlation. **D** The original data of the chip was converted to the log2 values after standardized processing, and a scatter plot was created using a two-dimensional coordinate system. Each point in the scatter plot represents a probe point on the microarray chip. The red dot indicates upregulated lncRNAs, and the green column indicates downregulated lncRNAs. The *X* and *Y* axes represent the signal values of samples 1 and 2, respectively. **E** Clinical validation of the ten up- and ten downregulated lncRNAs by qRT-PCR analysis. *Sn_CA*: cancerous tissue from sample n; *Sn_P_CA*: adjacent noncancerous tissue from sample *n*.

We used microarray to screen the lncRNA expression profiles of paired human PTC tumor and adjacent noncancerous tissue samples (4 vs. 4). After normalization of each sample, the boxplot demonstrated the good quality of this microarray (Fig. 1A). We evaluated Pearson’s correlation coefficient of total lncRNA expression level among different tissues, then draw heatmap and correlation plot of intersamples correlation (Fig. 1B, C). The difference in lncRNA expression levels was obvious between the tumor group and control group, but slight within each group. Finally, we found that 4337 lncRNAs were significantly differentially expressed between the PTC and noncancerous samples (*fc* > 1.5, *P* < 0.05). Among them, 1785 lncRNAs were upregulated and 2552 were downregulated in the PTC samples compared with the noncancerous samples which were represented by scatter plot (Fig. 1D). To verify the calculated data, 20 differentially expressed genes, including 10 upregulated and 10 downregulated genes were randomly selected for qRT-PCR in 60 pairs of PTC and adjacent noncancerous tissue samples. The results were consistent with the integrated data (Fig. 1E).

### PTC-associated lncRNA MREs predicted by miRcode

We chose the top fifteen dysregulated lncRNAs (12 upregulated, 3 downregulated) to construct the ceRNA network (*fc* > 5.0, *P* < 0.01) (Supplementary Table 2). Since miRNAs interact with lncRNAs through their MREs within the ceRNA network, we first searched for the potential MREs of lncRNAs. The MREs predicted by miRcode indicated that 85 miRNAs may interact with these fifteen lncRNAs (Supplementary Table 3). As an example, it was predicted that ENSG00000258068 (Gene symbol:RP11-328C8.5) could harbor miR-9, miR-9-3p, miR-204-5p.

### mRNAs targeted by miRNAs

To establish the ceRNA network, we next searched for miRNA–mRNA targets. Based on the miRNAs that might interact with lncRNAs, we searched TarBase for miRNA–mRNA targets with experimental support. The results showed that 28 miRNAs and 250 mRNAs were involved in the network and that most of these target genes are cancer-associated, which are listed in Supplementary Table 4. Their functions are involved in the cell cycle and cell proliferation, apoptosis, invasion and metastasis. The ceRNA regulatory networks may thus help explain the pathogenesis of papillary thyroid cancer.

### LncRNA-MIAT promotes the proliferation, migration, and invasion of PTC cells

**Fig. 2:**
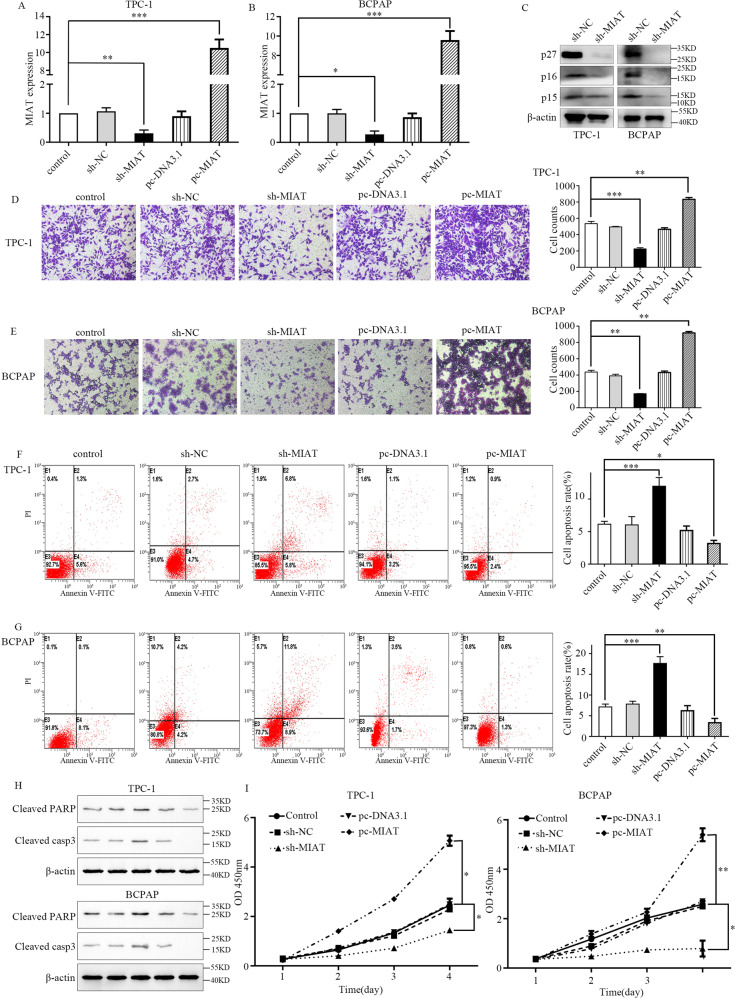
MIAT promotes the proliferation, migration, and invasion of papillary thyroid cancer cells. **A**, **B** MIAT expression was reduced in TPC-1 and BCPAP cells transfected with sh-MIAT, but increased in cells transfected with pcDNA3.1- MIAT. **C** The western blot of p27, p16, and p15. **D**, **E** The migratory abilities of TPC-1 and BCPAP cells were determined using transwell assay. **F**, **G** The apoptosis rate stained and analyzed by flow cytometry of TPC-1 and BCPAP cells were increased when MIAT were inhibited. E2, early apoptotic cells; E4, terminal apoptotic cells. The *Y* axis represents the apoptosis rate (E2 + E4). **H** The western blot of apoptosis-related protein including cleaved PARP and cleaved casp3 in TPC-1 and BCPAP cells. **I** TPC-1 and BCPAP cells were transfected with sh-NC/sh-MIAT or pcDNA3.1/pcDNA3.1- MIAT, cell viability was analyzed by CCK-8 assay. *P < 0.05, **P < 0.01, ***P < 0.001 compared with control cells, n.s. non-significant, using two-tailed *t*-test analysis.

The frequent upregulation of MIAT in the PTC tissues suggests that MIAT may play an important role in PTC progression. Thus, we performed gain- and loss-of-function experiments by transfecting respective vectors in TPC-1 and BCPAP. As shown in Fig. 2A and B, the sh-MIAT notably decreased MIAT expression, whereas pcDNA3.1-MIAT (pc-MIAT) significantly elevated MIAT expression. The western blot results demonstrated that MIAT knockdown led to the downregulation of p27, p16, and p15 (Fig. 2C). The migratory abilities of PTC cells were suppressed after downregulation of MIAT, which was revealed by a transwell assay (Fig. 2D, E). Flow cytometry demonstrated that MIAT downregulation accelerated apoptosis in PTC cell lines (Fig. 2F, G). Besides, apoptosis-associated proteins including cleaved PARP and cleaved CASP3 in TPC-1 and BCPAP were examined by western blot. The results showed that cleaved PARP and cleaved CASP3 were upregulated in the sh-MIAT group and downregulated in the pc-MIAT group, respectively (Fig. 2H). CCK-8 analysis displayed that knockdown of MIAT reduced the proliferation of TPC-1 and BCPAP cells (Fig. 2I).

### ceRNA network and lncRNA-MIAT regulate the expression of the miR-150-5p target gene EZH2

**Fig. 3:**
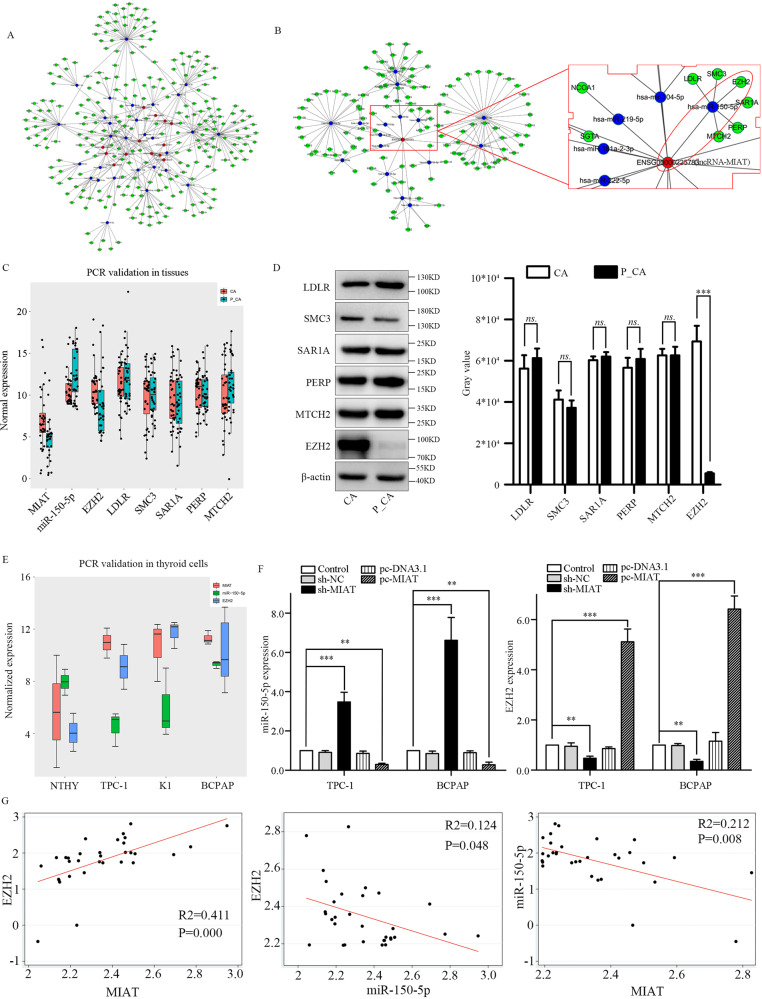
Interaction among three components of the ceRNA network. **A** An ceRNA network including 15 lncRNAs, 28 miRNAs, and hundreds of mRNAs were involved. The red dot represented the lncRNA, the blue dot represented the miRNA and the green ones represented the mRNA. **B** Construction of the ceRNA network demonstrated a relationship among lncRNA-MIAT, miR-150-5p, and EZH2. **C** MIAT, miR-150-5p and EZH2, LDLR, SMC3, SAR1A, PERP, MTCH2 expression in cancer tissue and para-cancer tissues. **D** The western blot of EZH2, LDLR, SMC3, SAR1A, PERP, MTCH2 in cancer and para-cancer tissues. **E** MIAT, miR-150-5p, and EZH2 expression in NTHY, TPC-1, K1, and BCPAP cells. **F** miR-150-5p and EZH2 expression in TPC-1 and BCPAP cells when sh-MIAT and pc-MIAT were used. **G** The Pearson’s correlation between lncRNA-MIAT, miR-150-5p, and EZH2 in PTC tissues. *P < 0.05, **P < 0.01, ***P < 0.001 compared with control cells, n.s. non-significant, using two-tailed *t*-test analysis.

Based on the above data, we constructed an ceRNA network. As shown in Fig. 3A, 15 lncRNAs and 28 miRNAs were involved in this ceRNA network. Through the ceRNA network, we found there may be a correlation among MIAT, miR-150-5p, and six mRNAs including LDLR, SMC3, EZH2, SAR1A, PERP, and MTCH2 (Fig. 3B). Next, we test the three components in clinical PTC samples and found that MIAT and EZH2 were highly expressed, miR-150-5p was low expressed, while other five mRNAs were no significant difference in cancer tissues (Fig. 3C). The western blot showed the same expression tendency (Fig. 3D). Then MIAT, miR-150-5p, and EZH2 were examined in NTHY, TPC-1, K1, and BCPAP cells to validate the reciprocal relationship between the three components. The results showed that the expression trend of MIAT and EZH2 demonstrated positively correlated, but negatively correlated with miR-150-5p (Fig. 3E). Knockdown of MIAT caused upregulation of miR-150-5p and downregulation of EZH2 in TPC-1 and BCPAP cells. Upregulation of MIAT showed the opposite changes (Fig. 3F). Next, the Pearson’s correlation between MIAT, miRNA-150-5p, and EZH2 in PTC tissues were calculated to validate the ceRNA correlation (Fig. 3G). These data suggest that MIAT upregulation contributes to PTC cell migration and proliferation and maybe partly due to the function of the ceRNA network.

### MIAT and EZH2 expression in TCGA

**Fig. 4:**
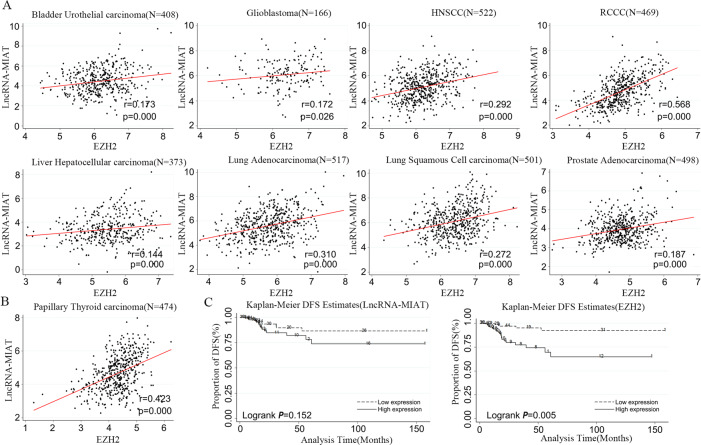
The correlation between lncRNA-MIAT and EZH2 validated by data from TCGA. **A** Regression analysis between lncRNA-MIAT and EZH2 in urothelial carcinoma, glioblastoma, head and neck squamous cell carcinoma (HNSCC), renal clear cell carcinoma (RCCC), hepatocellular carcinoma, lung adenocarcinoma, lung squamous cell carcinoma, and prostate adenocarcinoma. lncRNA-MIAT expression was positively correlated with EZH2 expression (*P* < 0.01). B Regression analysis between lncRNA-MIAT and EZH2 in PTC. **C** The Kaplan–Meier DFS estimate curves of lncRNA-MIAT and EZH2.

Our constructed ceRNA network shows that lncRNAs could interact with mRNAs in PTC. To confirm this finding, we performed a regression analysis using datasets from TCGA of other cancer types, including bladder urothelial carcinoma (*r* = 0.173, *P* = 0.000), glioblastoma (*r* = 0.172, *P* = 0.026), head and neck squamous cell carcinoma (*r* = 0.292, *P* = 0.000), renal clear cell carcinoma (r = 0.568, *P* = 0.000), hepatocellular carcinoma (*r* = 0.144, *P* = 0.000), lung adenocarcinoma (*r* = 0.310, *P* = 0.000), lung squamous cell carcinoma (*r* = 0.272, *P* = 0.000), and prostate adenocarcinoma (*r* = 0.187, *P* = 0.000). The results further confirmed that there existed a very good correlation between the ceRNA compositions (Supplementary Table 5 and Fig. 4A).

**Fig. 5:**
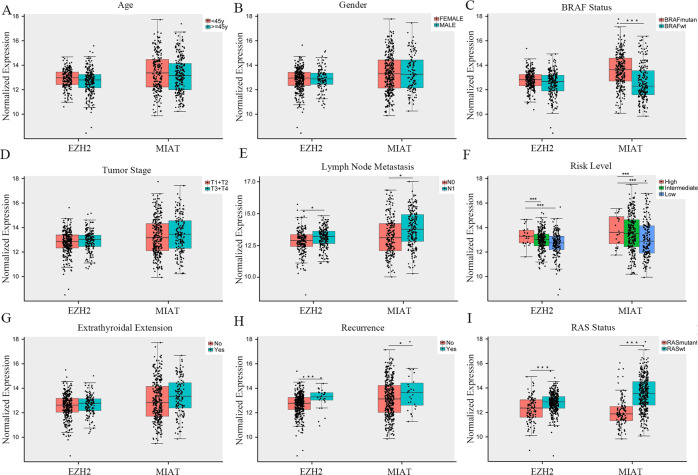
Association between lncRNA-MIAT and EZH2 expression and clinicopathological features in PTC. Significance differences in lncRNA-MIAT and EZH2 expression were found among various clinicopathological features, including age (**A**), gender (**B**), BRAF status (mutation vs. wild type) (**C**), tumor stage (T1 + T2 vs. T3 + T4) (**D**), lymph node metastasis (no vs. yes) (**E**), risk level (high vs. intermediate vs. low) (**F**), extrathyroidal extension (no vs. yes) (**G**), recurrence (no vs. yes) (**H**), and RAS status (mutation vs. wild type) (**I**). The X axis indicates lncRNA-MIAT and EZH2, the *Y* axis indicates normalized expression. The plots were created using the ggplot2 package of R. *P < 0.05, **P < 0.01, ***P < 0.001 compared with control cells, n.s. non-significant, using two-tailed *t*-test analysis.

We also found a good positive correlation between MIAT and EZH2 (*r* = 0.423, *P* = 0.000) (Fig. 4B). In addition, EZH2 was associated with the disease-free survival (DFS) of PTC patients (*P* = 0.005), while MIAT showed no significant associations(*P* = 0.152) (Fig. 4C). Furthermore, we calculated MIAT and EZH2 correlations for clinical factors, such as age, gender, BRAF status, RAS status, T stage, N stage, risk level, extrathyroidal extension, and recurrence (Supplementary Table 6 and Fig. 5). Upregulation of MIAT and EZH2 were positively related with LNM, high risk, recurrence and RAS mutation which indicates that MIAT and EZH2 may be played an important role in thyroid cancer progression.

### MIAT directly binds to and modulates miR-150-5p in PTC cells

**Fig. 6:**
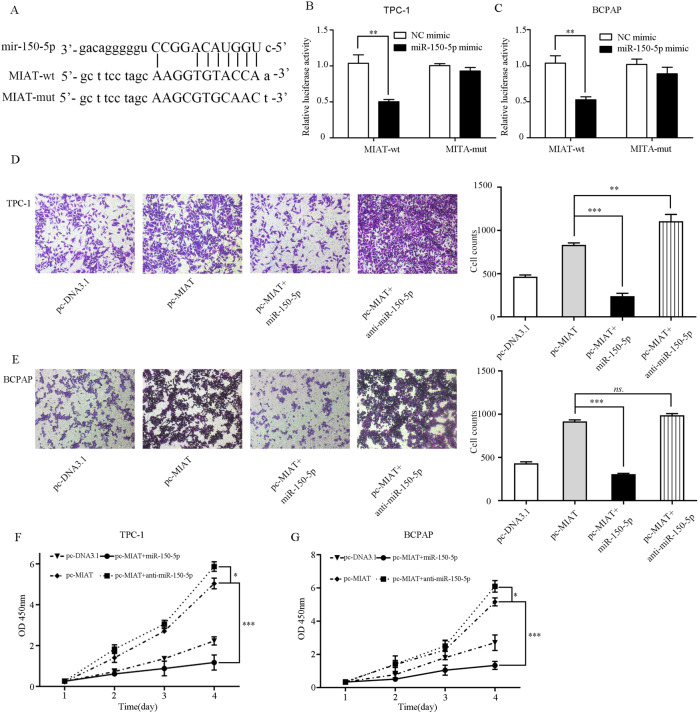
MIAT directly binds to and regulates miR-150-5p in PTC cells. **A** The binding sites of MIAT and miR-150-5p and the mutant sequence of MIAT based on binding region. **B**, **C** miR-150-5p mimic suppressed the luciferase activity of MIAT wild-type vector in TPC-1 and BCPAP cells. **D**, **E** The migration of cells was measured by transwell assay. **F**, **G** Cell viability was examined by CCK-8. *P < 0.05, **P < 0.01, ***P < 0.001 compared with control cells, n.s. non-significant, using two-tailed *t*-test analysis.

The target prediction tool Starbase showed that MIAT has the potential binding sites for miR-150-5p. The predictive binding sequence and the constructed mutant sequence are listed in Fig. 6A. Their relationship was further confirmed and we found that miR-150-5p mimic dramatically suppressed the luciferase activity of MIAT-wt, but not the luciferase activity of MIAT-mut compared to the control mimic in TPC-1 and BCPAP cells (Fig. 6B, C). We further investigated whether metastasis ability of thyroid cancer cells could function via mediating miR-150-5p in the situation with pcDNA3.1, pc-MIAT, pc-MIAT + miR-150-5p, and pc-MIAT + anti-miR-150-5p. The results demonstrated that the effects of MIAT on the migration (Fig. 6D, E) and proliferation (Fig. 6F, G) of TPC-1 and BCPAP cells were neutralized by anti-miR-150-5p.

### MIAT regulates EZH2 signaling by sponging miR-150-5p

**Fig. 7:**
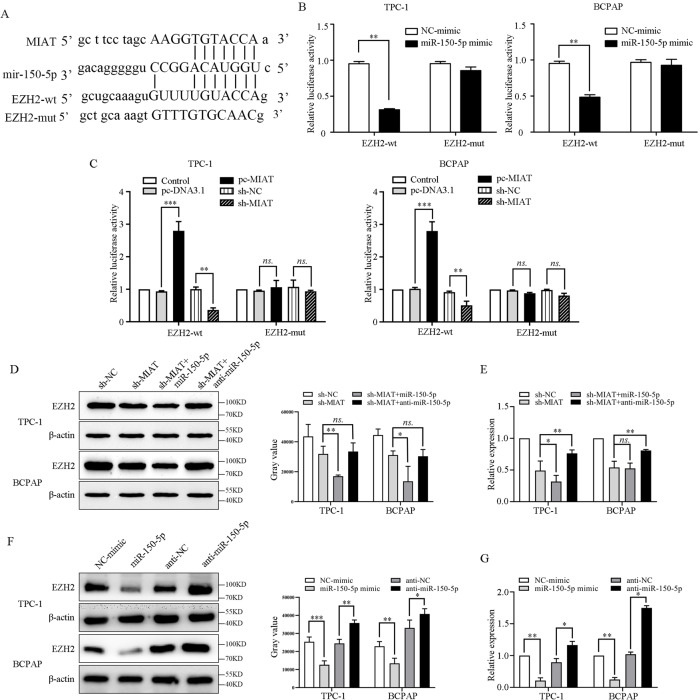
MIAT regulates EZH2 expression by sponging miR-150-5p in papillary thyroid cancer. **A** The binding sites of miR-150-5p and EZH2, and the mutant sequence of EZH2based on the binding region. **B** miR-150-5p mimic suppressed the luciferase activity of EZH2 wild-type vector in TPC-1 and BCPAP cells. **C** pc-MIAT enhanced and sh-MIAT decreased the luciferase activity of EZH2 wild-type vector in TPC-1 and BCPAP cells. **D** Protein levels of EZH2 in TPC-1 and BCPAP cells were determined by western blot when regulate the expression of MIAT. **E** Protein levels of EZH2 in TPC-1 and BCPAP cells were determined by western blot when regulate the expression of miR-150-5p. **F** Relative expression of EZH2 in TPC-1 and BCPAP cells transfected with sh-NC, sh-MIAT, sh-MIAT + miR-150-5p, and sh-MIAT + anti-miR-150-5p by PCR. **G** Relative expression of EZH2 in TPC-1 and BCPAP cells transfected with NC-mimic, miR-150-5p mimic, anti-NC, and anti-miR-150-5p. *P < 0.05, **P < 0.01, ***P < 0.001 compared with control cells, n.s. non-significant, using two-tailed *t*-test analysis.

The potential binding sites of miR-150-5p and EZH2 and the EZH2 mutant sequence are listed in Fig. 7A. The luciferase reporter assay showed that miR-150-5p mimic obviously inhibited the luciferase activity of EZH2-wt, while had no influence on that of EZH2-mut TPC-1 and BCPAP cells (Fig. 7B). Upregulation of MIAT prominently increased the luciferase activity of EZH2-wt and vice versa. Similarly, there were no significant effects on EZH2-mutin when modulating MIAT (Fig. 7C). The western blot showed that knockdown of MIAT reduced EZH2 expression, and further strengthened by miR-150-5p mimic (Fig. 7D). The PCR demonstrated that the downregulation of EZH2 by sh-MIAT attenuated by anti-miR-150-5p (Fig. 7E). Furthermore, both the western blot and PCR revealed that miR-150-5p mimic notably decreased EZH2 expression, while anti-miR-150-5p rescued the expression of EZH2 both in TPC-1 and BCPAP cells (Fig. 7F, G).

### MIAT downregulation decreases tumor growth in vivo that is related to the miR-150-5/EZH2 axis

**Fig. 8:**
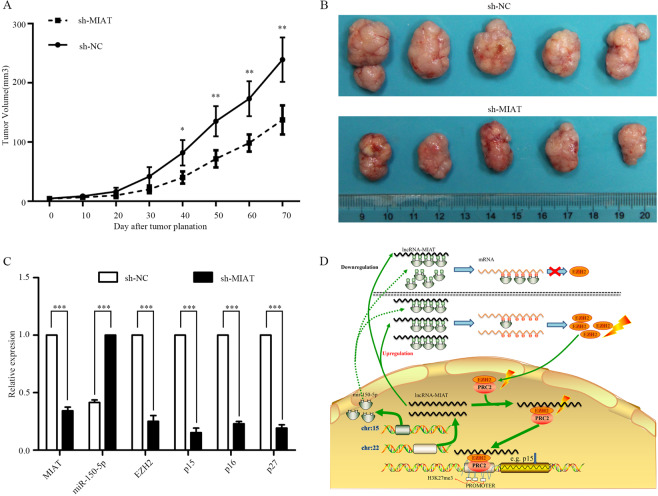
MIAT inhibited tumor growth in vivo and the hypotheses of the ceRNA. **A**, **B** sh-NC and MIAT-shRNA of TPC-1 cells stably injected subcutaneously into different groups of nude mice. Tumor volume was measured with Vernier calipers every 4 days, and calculated as a (length) × b^2^ (width). Five weeks after the injection, mice were photographed and killed, tumor growth curves were obtained. Error bars indicate mean ± SE/SD, compared with the control group. **C** qRT-PCR analyses of tumor issues showed that the mRNA and protein expression of the relative components decreased. **D** Molecular mechanism hypotheses of MIAT on the biological behavior of papillary thyroid carcinoma cells. MIAT and hsa-mir-150-5p were encoded by 22q12.1 and 19q13.33, respectively. Upregulation of MIAT combined with more miRNA (e.g., hsa-mir-150-5p etc.), resulting in the decrease of free hsa-mir-150-5p, the number of proteins encoded by EZH2 is relatively increased, as well as the EZH2/PRC2 complex. The complex can combine with the promoter H3K27me3 structure of tumor suppressor genes such as p15, and inhibit the expression of tumor suppressor genes, so as to promote the proliferation, invasion and metastasis of PTC cells. *P < 0.05, **P < 0.01, ***P < 0.001 compared with control cells, n.s. non-significant, using two-tailed t-test analysis.

To determine whether MIAT could influence the biological functions of thyroid cancer, MIAT expression was stable knockdown by transfection with shRNA and sh-NC and inoculated to nude mice. The results showed that tumors grown from MIAT stable knockdown cells were slower than tumors grown from the sh-NC cells (Fig. 8A, B). The mice weight of the sh-MIAT group was not significant to the sh-NC group (Supplementary Fig. 1). PCR assays showed that the MIAT expression levels were downregulated in tumor tissues collected from the sh-MIAT group compared with the controls, and the relative downstream components including EZH2, p16, p15, and p27 decreased significantly (Fig. 8C). These data suggest upregulation of lncRNA-MIAT contributes to PTC cell migration and proliferation maybe partly due to the function of the MIAT-150-5p-EZH2 network. The hypothesis is illustrated in Fig. 8D.

## Discussion

The prognosis of patients with PTC is generally favorable because surgery, with or without radioactive iodine therapy, can achieve complete remission in most cases [10]. However, the current diagnostic and therapeutic strategy are not completely effective. Over the past few decades, researchers have determined that PTC tumorigenesis is a complex biological process characterized by various molecular abnormalities, such as BRAFV600E and TERT promoter mutations, as well as mutations in RET/PTC, PAX8/PPARα, and galection-3 among others [11–14]. However, the exact pathogenesis of this disease remains unclear.

ncRNAs, including miRNAs and the lncRNAs, have been proposed in the past decade to act as regulators of cancer pathways and serve as predictive biomarkers of cancer prognosis [15]. Although the functions of lncRNAs remain under debate, certain lncRNAs have been found to have functions related to the regulation of gene expression, at the levels of post-transcriptional processing, RNA maturation, and transport [16].

In this study, by constructing a lncRNA–miRNA–mRNA network, we found that lncRNA-MIAT might play an important role in the development of PTC via interactions with mir-150-5p, which then regulates EZH2 signaling.

MIAT is a lncRNA that was initially identified to be associated with myocardial infarction [17], but recent studies have implicated that MIAT is also involved in paranoid schizophrenia [18], diabetes-related diseases [19, 20], neurovascular dysfunction [21], cardiac hypertrophy [22], and cancer [23, 24]. MIAT has been shown to be highly expressed in diabetic retinas and endothelial cells [25, 26] cultured in a high-glucose medium. MIAT silencing significantly inhibits endothelial cell proliferation and migration. Researchers have found that the MIAT/miR-29b/Sp1 and MIAT/miR-22-3p/DAPK2 networks appear to be important in this process [19]. Qu et al. elucidated the cellular function and pathological role of MIAT in the heart: their results suggest that the MIAT/miR-24/Furin/TGF-β1 network participates in the system controlling cardiac fibrosis [22]. Mechanistically, MIAT functions as a ceRNA network component to regulate VEGF/Akt levels by sponging miR-150-5p in retinal endothelial cells [21, 27]. Recent results from Sattari et al. showed that MIAT has potential as a new biomarker for the aggressiveness of chronic lymphocytic leukemia [28]. In addition, the MIAT/miR-150-5p/ZEB1 signaling pathway plays an oncogenic role in non-small cell lung cancer [29]. All of these results indicate that MIAT is a cancer-promoting gene.

EZH2 is another key factor in our study. It is one of the core components of polycomb repressive complex 2 (PRC2), which functions as a lysine methyl-transferase, especially to catalyze the tri-methylation of histone 3 at lysine 27 (H3K27me3) [30]. Elevated EZH2 expression is well recognized in a wide range of cancers, including PTC, and its expression is strongly linked to tumor malignancy, invasiveness, and poor prognosis [31–33]. ncRNAs have been demonstrated to modulate the level of EZH2 through post-transcriptional mechanisms directly or indirectly.

BRAF-activated lncRNA (BANCR) is enriched by polycomb EZH2, and silencing BANCR leads to decreased chromatin recruitment of EZH2, resulting in significantly reduced thyroid-stimulating hormone receptor expression [34]. In Gastric Cancer, LINC00673 regulates Oncogenic biological behavior by Interacting with LSD1 and EZH2 [35]. Upregulating SNHG20 promotes cell proliferation and migration by epigenetically silencing of P21 expression through EZH2 in non-small cell lung cancers [36]. In contrast, miRNAs can bind to EZH2 RNA transcripts and regulate their stability, integrity, and translation, thus directly affecting the protein expression of EZH2. For example, miR-101 decreases the invasion and migration ability of several tumor types, including osteosarcoma in vitro and gastric cancer and glioblastoma in vitro and in vivo, through the post-transcriptional downregulation of EZH2 [37–39]. Moreover, miR-26a, miR-138, and miR-124 also participate in the post-transcriptional regulation of EZH2 in different types of cancer cells [40–42]. Collectively, these observations are the basis for our conjecture regarding the relationship among lncRNA-MIAT, mir-150-5p, and EZH2.

In summary, this study demonstrates a significantly altered lncRNA expression profile in PTC. Dysregulation of these lncRNAs may be important in the development, invasion, and metastasis of tumors. To the best of our knowledge, these are the first findings to demonstrate the biological function of MIAT in PTC. Our study provides novel insight into the role of MIAT in cancer and may aid in the development of diagnostic and therapeutic tools for thyroid cancer treatment and diagnosis.

## Supplementary information

supplementary files

Detailed Attribution of Authorship

Reproducibility Checklist forms

## Data Availability

The data that support the findings of this study are available from the corresponding author upon reasonable request.
