# Supplementary material for: LncRNA-MIAT promotes thyroid cancer progression and function as ceRNA to target EZH2 by sponging miR-150-5p
Source: Cell Death Dis. 2021 Nov 22;12(12):1097. doi: 10.1038/s41419-021-04386-0 (PMC8608816; doi:10.1038/s41419-021-04386-0)
Supplement: Supplementary file 1 — supplementary files [file 41419_2021_4386_MOESM1_ESM.pdf]

Supplementary Figure 1. The weight of nude mice which injected subcutaneously into sh-NC and MIAT-shRNA of TPC-1 cells. Error bars indicate mean ± SE/SD, compared with the control group.

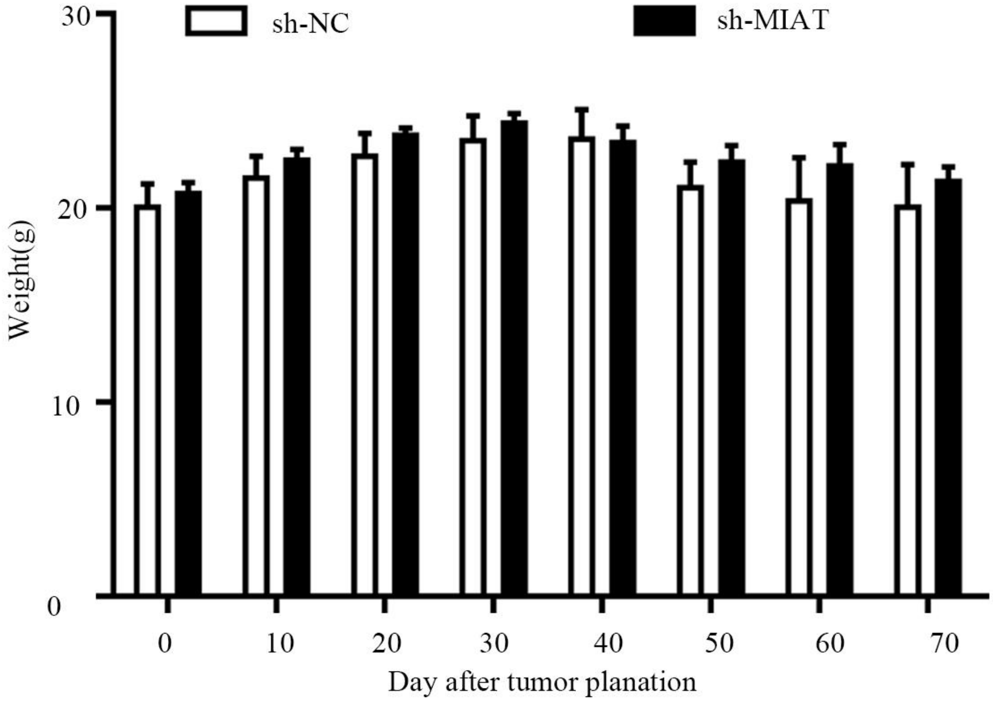

Supplementary table 1. Antibody information.

REAGENT	SOURCE	IDENTIFIER
**Anti-p27**	Proteintech	67355-1-Ig
**Anti-p16**	Proteintech	10883-1-AP
**Anti-p15**	abcam	Ab53034
**Anti-Cleaved PARP**	abcam	Ab32064
**Anti-Cleaved casp3**	abcam	Ab2302
**Anti-LDLR**	Proteintech	66414-1-Ig
**Anti-SMC3**	Proteintech	14185-1-AP
**Anti-SAR1A**	Proteintech	22291-1-AP
**Anti-PERP**	abcam	ab5986
**Anti-MTCH2**	Proteintech	16888-1-AP
**Anti-EZH2**	Proteintech	66476-1-lg
**Anti-β-actin**	Proteintech	66009-1-Ig

Supplementary table 2. A collection of top 15 differentially expressed lncRNAs between PTC cancer tissues and paracancerous tissues.

	Ensemble	GeneSymbol	Dysregulation	*P* values	Foldchanges
**1**	ENSG00000258068	RP11-328C8.5	up	0.000	7.269
**2**	ENSG00000256234	RP11-283G6.4	up	0.008	13.379
**3**	ENSG00000254417	ANO1-AS2	up	0.001	7.7381
**4**	ENSG00000250748	RP11-230G5.2	up	0.004	22.939
**5**	ENSG00000237265	RP11-402P6.9	up	0.004	7.863
**6**	ENSG00000236481	AC002331.1	up	0.006	5.005
**7**	ENSG00000227911	RP11-141M1.1	down	0.008	0.189
**8**	ENSG00000226005	RP11-464C19.3	down	0.004	0.195
9	***ENSG00000225783***	***MIAT***	***up***	***0.009***	***5.891***
**10**	ENSG00000225511	LINC00475	up	0.005	6.554
**11**	ENSG00000224568	AC096669.3	down	0.008	0.121
**12**	ENSG00000223914	AC079630.2	up	0.003	19.380
**13**	ENSG00000214797	RP11-1036E20.9	up	0.006	6.869
**14**	ENSG00000206120	EGFEM1P	up	0.009	11.388
**15**	ENSG00000171889	MIR31HG	up	0.003	10.308

Supplementary table 3. Possible miRNAs targeting lncRNA.

lncRNA	miRNA
**ENSG00000258068**	miR-9, miR-9-3p, miR-204-5p
**ENSG00000256234**	miR-96, miR-96-5p, miR-137, miR-138-1, miR-138-1-3p, miR-138-2, miR-138-5p, miR-182-5p, miR-182
**ENSG00000254417**	miR-9-3, miR-9-3p, miR-9, miR-106, miR-20b, miR-106b, miR-613, miR-204-5p, miR-375, miR-125a-5p
**ENSG00000250748**	miR-9-3, miR-9-3p, miR-9, miR-106, miR-106b, miR-135b-5p, miR-138-1, miR-138-1-3p, miR-138-2, miR-138-5p, miR-141, miR-200a, miR-146, miR-146a, miR-146b, miR-146b-3p, miR-146-5p, miR-15a, miR-16, miR-20b, miR-181a, miR-181c, miR-181b-5p, miR-181a-2-3p, miR-182, miR-182-5p, miR-193, miR-196b-5p, miR-613, miR-203a, miR-21, miR-21-5p, miR-21-3p, miR-24-1, miR-24-2, miR-25-3p, miR-26a-1, miR-26a, miR-101, miR-31, has-miR-31-5p, miR-124-3p, miR-375
**ENSG00000237265**	miR-96, miR-96-5p, miR-137, miR-138-1, miR-138-2, miR-138-5p, miR-138-1-3p, miR-146, miR-146a, miR-146b, miR-146b-5p, miR-146a, miR-146b-3p
**ENSG00000236481**	miR-106, miR-96, miR-96-5p, miR-182, miR-182-5p
**ENSG00000227911**	miR-138-1, miR-138-2, miR-138-5p, miR-138-1-3p, miR-199b, miR-199a-3p, miR-25-3p, miR-26a-1, miR-26a
**ENSG00000226005**	miR-199b, miR-24-1, miR-24-2
**ENSG00000225783**	miR-130a-3p, miR-9-3, miR-9-3p, miR-9, miR-106, miR-96, miR-96-5p, miR-141, miR-200a, miR-155, miR-155-5p, miR-15a, miR-16, miR-20b, miR-106, miR-181a, miR-181c, miR-181b-5p, miR-181a-2-3p, miR-182, miR-182-5p, miR-187-3p, miR-196b-5p, miR-199b, miR-613, miR-203a, miR-204-5p, miR-219-1, miR-219-5p, miR-221, miR-221-5p, miR-221-3p, miR-222, miR-222–5p, miR-222-3p, miR-24-1, miR-24-2, miR-25-3p, miR-29c, miR-29b, miR-29a-2, miR-29, miR-29a, miR-34a, miR-34, miR-34a-5p, miR-449, miR-125a-5p, miR-150-5p
**ENSG00000225511**	miR-96, miR-96-5p, miR-137, miR-141, miR-200a, miR-15a, miR-16, miR-182, miR-182-5p, miR-183, miR-183-5p, miR-203a, miR-204-5p, miR-29a, miR-29a-2, miR-29b, miR-29c, miR-30a-5p, miR-124-3p, miR-34a, miR-34, miR-34a-5p, miR-125a-5p
**ENSG00000224568**	miR-141, miR-106, miR-106b, miR-187-3p, miR-204-5p, miR-221, miR-221-5p, miR-221-3p, miR-222, miR-222-3p, miR-222-5p, miR-101, miR-34a, miR-449, miR-125a-5p
**ENSG00000223914**	miR-141, miR-200a, miR-199b, miR-203a, miR-219-5p, miR-34a, miR-34, miR-449, miR-375
**ENSG00000214797**	miR-137, miR-141, miR-200a, miR-146a, miR-146b, miR-146b-5p, miR-155, miR-155-5p, miR-20b, miR-106, miR-106b, miR-203a, miR-204-5p, miR-219-1, miR-219-5p, miR-24-1, miR-24-2, miR-25-3p, miR-31, miR-31-5p, miR-124-3p, miR-34, miR-34a, miR-449, miR-125a-5p, miR-451, miR-451a
**ENSG00000206120**	miR-130a-3p, miR-130b-5p, miR-106, miR-96, miR-96-5p, miR-135b-5p, miR-138-1, miR-138-2, miR-138-5p, miR-138-1-3p, miR-141, miR-200a, miR-15a, miR-16, miR-20b, miR-106, miR-106b, miR-181c, miR-181a, miR-181b-5p, miR-181a-2-3p, miR-182, miR-182-5p, miR-183, miR-183-5p, miR-193, miR-199b, miR-221, miR-221-5p, miR-221-3p, miR-222, miR-222-3p, miR-222-5p, miR-24-1, miR-24-2, miR-26a, miR-26a-1, miR-29, miR-29a, miR-29b, miR-29c, miR-29a-2
**ENSG00000171889**	miR-193, miR-613, miR-34a, miR-34, miR-449

Supplementary table 4. Validated mRNAs' targets from TarBase.

miRNA	mRNA
miR-96-5p	FRS2, CREB3L2, SPEN, ANKRD52, ZIC2, HBP1, EIF4EBP2, ANKIB1, SESN3, LEPR, BCL2, KDM5A, WWP1, EEA1, FAM110B, RECK
miR-375	NIPBL, PLEKHA3, YOD1, DCP2
miR-34a-5p	E2F3, SGTA, ANKRD52, PEG10
miR-31-5p	HIF1AN, LDOC1L, PANK3, HIAT1, NUFIP2, HIPK1, FOXC1
miR-30a-5p	PDE7A, CCNE2, PLAGL2, ERLIN1, RHOB, SPEN, CDC37L1, LRCH2, JOSD1, ANKRA2, CEP350, LRRC8D, TAOK1, UBE3C, UBN1, ZNF711, SEC23A, SMARCD2, RAPGEF2, RBM12, TBPL1, DPYSL2, MARCH6, BIRC6, ZFAND5, WDR26, ZBTB18, CPE, SLC35C1, NAPG, GXYLT1, CBX2, CPEB4, KMT2A, SLC35B4, IGF1R, NRBP1
miR-25-3p	HIPK3, SOX4, FAM20C, MIA3, BTG2, CD2AP, MAN2A1, SFXN1, TOB1, GFPT2, KIF5B, FNDC3B, GOLGA3, INSIG1, IRS2, KIAA1279, SIK1, MAP1B, DNAJB12, GATA6, DNAJC27, MYLIP, SLX4, FAM160B1, PHTF2, CAND1, NPTX1, CCT6A
miR-222-5p	UTRN
miR-222-3p	ZFYVE16, YWHAG, WSB2, VAPB, MIDN
miR-221-3p	DDX3X, WSB2, VAPB, ZFYVE16, YWHAG, TRPS1, MIDN
miR-219-5p	NCOA1
mir-204-5p	PRPF38B, ZNF704
miR-21-3p	MAT2A, GPM6A, NR6A1, PDE4D, ZNRF1, ERCC6, IGF2R
mir-150-5p	LDLR, PERP, SMC3, SAR1A, MTCH2, EZH2
miR-203a	AFF4, XRN2, NFYA, ATP11A, CUL1
miR-199a-3p	ZHX1, TFAM, SLC20A2, CEP350, KIF5B, HIPK3
miR-196b-5p	HOXC8, SLC9A6, HAND1, HOXA9, PSMD11, GATA6, HOXB6, IGF2BP1, PTPRG, PRTG, YOD1, EPHA7, CCDC126
miR-183-5p	LRP6, PLAGL2, MAPK1IP1L, EEF2, LAPTM4A
miR-182-5p	FRS2, MBNL2, RECK, WWP1, PHF13, BCL2, SESN3, KDM5A, ARCN1, RAB3GAP2, ANKRD52, NUFIP2, ZHX2, EIF3H, CEBPA, HBP1, CREB3L2
miR-181b-5p	BIRC6, CCNJ, PKD2, DYNC1LI2
miR-181a-2-3p	SETD7, ZNF264, MZT1, KLHL15, NLK, TIA1
miR-155-5p	JARID2, E2F2, RNF19A, RPS20, DSG2, RSF1
miR-146b-3p	MARCH9, NUFIP2, CMPK1
miR-138-5p	RMND5A, SOGA1, CCNE1, SEMA4C, CAMK2N1, RPL28, NCL, THRAP3, OSTM1, KDM5A, SETBP1, GLCCI1, MLEC, DDX3X,
miR-138-1-3p	ZFP36L2, DUSP5, SLMAP, BAG4, LNPEP, XPOT, TNPO1, PNISR, FBXO38
miR-137	PITPNA, CD2AP, MYO1C, XRN1, SBNO1, IRS2, GOLGA3, FNIP2, PAPD7, CALM3, MIA3, MAN2A1, H3F3B, PDZD2, ADAMTS5, KCNC4, SP1, DEMND4B, HNRNPU, RRAGD, GPR180, GCLC, SFXN1, UBE3C, FAM20C, AZIN1, AGO1, ZNF770, HOXC8, STK39, MKRN2, SLX4, TMEM245, VLDLR, DNAJB12, NFIC, SLC30A7, CCT6A, HAND1, MTHFD2
miR-135b-5p	CEP85L, SLC6A5, LATS2, RB1CC1, DDX3X, CENPB
miR-130a-3p	B4GALT5, PPP6R1, TSHZ1, PSAP, C7orf60, LDLR, ARL6IP1, SMARCD2, SESTD1, PTPN4, MLLT10, PGM2L1, ZMAT3, ARAP2, ANKRD52, FAM178A, HPRT1, STEAP4, CNOT4, ATP11A, SPTY2D1, PPP1R15B
miR-124-3p	VAMP3, CD164, RAB10, TARBP1, HIPK3, LAMC1, RRAGD, PTBP1, SLC35F5, QSER1, DCAF16, STK35, CGN, AGO1, KDELR2, MAPRE1, HOXA5

Supplementary table 5. Validation of relation between LncRNA-MIAT and EZH2 in kinds of carcinomas from TCGA.

Cancer Types	Objectives	Spearman's Rho	*P*
**Bladder Urothelial carcinoma**	408	0.173	0.000
**Glioblastoma**	166	0.172	0.026
**Head and neck Squamous Cell carcinoma**	522	0.292	0.000
**Kidney Renal Clear cell carcinoma**	469	0.568	0.000
**Liver Hepatocellular Carcinoma**	373	0.145	0.005
**Lung Adenocarcinoma**	517	0.310	0.000
**Lung Squamous Cell carcinoma**	501	0.272	0.000
**Prostate Adenocarcinoma**	498	0.188	0.000
**Papillary Thyroid Carcinoma**	496	0.424	0.000

Supplementary table 6. Relationship between MIAT/EZH2 expression and clinicopathologic factors of patients with PTC.

Parameter	No. of patients	LncRNA-MIAT		EZH2	
		z	*P* value	*z*	*P* value
**Age**					
<45y *vs.*>=45y	211/243	-1.42	0.154	-1.11	0.269
**Gender**					
Female *vs.*Male	331/123	0.26	0.791	0.820	0.415
**BRAF status**					
Mutant *vs.* Wild type	267/207	4.04	***0.000***	1.76	0.079
**Tumor stage**					
T3+T4 *vs.* T1+T2	282/169	0.76	0.448	0.93	0.351
**N stage**					
N1*vs.*N0	207/244	2.05	***0.041***	2.50	***0.031***
**Risk level**					
High *vs.*Intermediate *vs.*Low	166/252/24	12.802	***0.002***	16.162	***0.000***
**Extrathyroidal extension**					
Yes *vs.*No	132/309	0.62	0.538	0.45	0.651
**Recurrence**					
Yes *vs.*No	27/382	2.08	***0.038***	3.03	***0.002***
**RAS status**					
Mutant *vs.*Wild type	118/356	-5.90	***0.000***	-4.11	***0.000***
