# Supplementary material for: LncRNA-MIAT promotes thyroid cancer progression and function as ceRNA to target EZH2 by sponging miR-150-5p
Source: Cell Death Dis. 2021 Nov 22;12(12):1097. doi: 10.1038/s41419-021-04386-0 (PMC8608816; doi:10.1038/s41419-021-04386-0)
Supplement: Supplementary file 2 — Detailed Attribution of Authorship [file 41419_2021_4386_MOESM2_ESM.pdf]

DECLARATION OF CONTRIBUTIONS TO ARTICLE **ADMC**

Manuscript Number: CDDIS-20-4806

Journal Name: *Cell Death & Disease* (the 'Journal')

Proposed Title of the Contribution:

LncRNA-MIAT promotes thyroid cancer progression and function as ceRNA to target EZH2 by sponging miR-150-5p (the 'Contribution')

Author(s):

Guo, Kai; Qian, Kai; Shi, Yuan; Sun, Tuanqi; Wang, Zhuoying (the 'Authors')

For all *CDDis* articles, each person named as an author in the published version must be able to show he or she has contributed substantially to the article.

Authorship credit should be based on 1) substantial contributions to conception and design, acquisition of data, or analysis and interpretation of data; 2) drafting the article or revising it critically for important intellectual content; and 3) final approval of the version to be published. Authors should meet conditions 1, 2 and 3.

Any person who cannot be shown to have made a substantial contribution to the article cannot be listed as an author in the final version. The name of any person who is deemed to have made a minor contribution can, however, appear in the Acknowledgments section of the article.

Please complete the table below to indicate the contributions of all named authors to the manuscript.

Author Full Name:	Specification of Contribution to the Manuscript:
Guo, Kai	Guo, Kai analyzed and interpreted the data and wrote the article including the figures and tables.
Qian, Kai	Qian, Kai analyzed and interpreted the data and wrote the article including the figures and tables.
Shi, Yuan	Shi, Yuan prepared the materials and performed the experiment.
Sun, Tuanqi	Sun, Tuanqi critical revised the manuscript for important intellectual content and supervised the entire work.
Wang, Zhuoying	Wang, Zhuoying critical revised the manuscript for important intellectual content and supervised the entire work.

扫描全能王 创建

Please complete the table below to indicate the contributions of all named authors to the figures.

Figure 1:

Guo Kai and Qian Kai analyzed the microarray data and drew the Box plot, Heatmap, Correlation plot and Scatter Plot (Figure 1A-D). Shi, Yuan collected the cancer and paracancer tissues and performed the PCR experiments (Figure 1E), and the column plot were generated by Guo Kai using Graphpad.

Figure 2:

The PCR of knockdown and overexpress and western blot were performed by Guo Kai (Figure 2A-C and H). The transwell, apoptosis and CCK-8 were performed by Shi Yuan (Figure 2D-G and I).

Figure 3:

The ceRNA network produced using cystoscope (Figure 3A-B) and PCR, western blot was demonstrated by Guo Kai (Figure 3C-D). The MIAT, miR-150-5p and EZH2 expression in cells and relative expression in different status of MIAT were performed by Shi Yuan (Figure 3E-F). The correlation of three components were drew by Qian Kai (Figure 3G).

Figure 4:

The correlation scatter plot between MIAT and EZH2 in kinds of cancers (Figure 4A) and K-M plot (Figure 4B) were drew by Qian Kai.

Figure 5:

The expression of MIAT and EZH2 between different clinical factor groups were performed by Guo Kai using R project (Figure 5A-I).

Figure 6:

The dual luciferase reporter assay was performed by Guo Kai (Figure 6A-C). The transwell and CCK-8 were performed by Shi Yuan (Figure 6D-G).

Signed for and on behalf of the Author(s):	Print Name:	Date:
Guo, Kai	Guo, Kai	2021.06.20

扫描全能王 创建
